# Prevalence and risk factors associated with the comprehensive needs of cancer patients in China

**DOI:** 10.1186/s12955-019-1171-4

**Published:** 2019-06-13

**Authors:** Xin-Shuang Zhao, Hong-Yun Wang, Luo-Ling Zhang, Yan-Hua Liu, Hai-Yan Chen, Ying Wang

**Affiliations:** 1 College of Nursing, Henan University of Science and Technology, Luoyang, 471023 People’s Republic of China; 2 The 1st Affiliated Hospital of Henan University of Science and Technology, Luoyang, 471023 People’s Republic of China; 3 Henan University of Science and Technology, Luoyang, 471023 People’s Republic of China

**Keywords:** Cancer, Patient, Need, Factor

## Abstract

**Background:**

The incidence and mortality rates of cancer have been increasing in developing countries, particularly in Asia. Therefore to provide optimal comprehensive care to the cancer patients, the care plan must focus on the comprehensive needs of cancer patients. The purpose of this study was to investigate the comprehensive needs of cancer patients, and explore the associated factors.

**Methods:**

In a cross-sectional questionnaire study, a total of 200 cancer patient-caregiver dyads were selected and interviewed in Mainland China by convenient sampling method. Patients’ comprehensive needs were assessed with Comprehensive Needs Assessment Tool in cancer for Patients (CNAT), including seven domains (Information, Psychological Problems, Health Care Staffs, Physical Symptoms, Hospital Facilities and Services, Social/Religious/Spiritual Support and Practical Support). Both cancer patients and caregivers completed the sociodemographic survey. The mean differences in domain scores for different characteristics groups were compared by one-way ANOVA or non-parametric analyses, and influencing factors defined with multivariate regression analysis.

**Results:**

The cancer patients’ need for Health Care Staffs (78.35 ± 13.08) was the highest among the seven domains, followed by the need for Information (71.18 ± 17.39) and the need for Hospital Facilities and Services (52.65 ± 13.35). The lowest score was the need for Physical Symptoms (35.12 ± 16.68). Patients who were female, with low family monthly income, at their own expense, and with highly educated caregivers had higher score of CNAT. Also sociodemographic characteristics were associated with each domain need of cancer patients.

**Conclusion:**

This study shows that cancer patients experience high levels of needs for health-care staff and information, and the different needs are closely related to their sociological characteristics. The provision of health care can be adapted to meet the different needs of cancer patients of different epidemiological characteristics at different times during the course of treatment.

## Background

Recently the cancer incidence and mortality rates have been increasing in developing countries, particularly in Asia [1, 2], making cancer the leading cause of death and a major public health problem [3]. According to the latest study, the incidence rates of cancers such as lung and colorectal in some Asian countries have surpassed those of Western countries [4]. With the development of cancer detection and treatment, the number of patients diagnosed with cancer is increasingly growing [5]. While a cancer diagnosis is often a sudden major event to most of patients and the family, and often sparks an abrupt need for diagnostic and treatment decisions as well as active involvement by both the patients and the family [6].

Because of the increasingly advanced treatment methods, more patients live a long period with a diagnosis of cancer, which makes cancer a big problem with continuous care [7]. As the life-threatening illness cancer is a serious challenge and a heavy stress to the patients, it would be difficult, challenging and exhausting for cancer patients to cope with sudden disease [7, 8]. And as a result, the cancer patients suffer from the disease burden, such as physical discomfort, mental stress and economic pressure, associated with the symptoms and treatment of cancer [9, 10]. The care activities of cancer patients include estimating, planning, decision making, symptom assessment, problem solving, and accessing health system. This complex care of cancer patients needs comprehensive knowledge and skills while many patients are uncertain about the concrete situation and severity of disease, survival time, how to improve the condition, and how to adjust the psychological pressure [11].

Patient-centred care is the gold standard for provision of healthcare in the world [12]. It need to be recognized that cancer patients have legitimate needs for help from health care professionals and the social support. Recently, the International Psycho-oncology Society (IPOS) published its Standard of Quality Cancer Care [13], a new quality standard to support the development and implementation of new clinical practice guidelines. Accordingly, care should no longer focus solely on delivery of medical treatment but also look to encompass the person’s needs for information, practical support, psychological and social support in order to fully support that person’s physical, emotional and psychological wellbeing throughout their illness. And if the needs of patients were not met, it may have impact on their mental and physical health, and consequently patients may miss the disease recovery.

Therefore to provide optimal comprehensive care to the cancer patients, the care plan must focus on the comprehensive needs of cancer patients [14]. In terms of symptom management and pressure adjustment, the majority of patients needed substantial help, however that need was unmet for in many previous studies [15, 16]. These results are directly linked to the patients’ quality of life, personal aspirations, values, and quality of their relations and needs. For healthcare staffs, assessment of these various needs means that they need to pay more attention to the patients themselves as individuals during the various stages of their disease, beginning from diagnosis until the terminal phase.

Although many studies have investigated the prevalence of the unmet needs among various types of cancer patients in Asian countries, only the information need, psychosocial need or some unilateral need was explored, or just one particular type of cancer, lack of comprehensiveness [17–19]. Several factors associated with high care needs had been confirmed by some previous studies, such as socio-demographic factors (age, gender, education level, marital status or financial situation), and clinical factors (sick time, severity, type of treatment received and physical function) [7, 8, 20]. Early detection of the comprehensive needs of cancer patients is important not only to reduce the suffering caused to the patient but it may improve the quality of cancer care [21].

So it is very important to understand the comprehensive needs of cancer patients for developing and improving services to address the identified gaps in cancer care [22]. The original scales were developed for cancer patients and caregivers, of which one was CNAT (for patients) and the other was CNAT-C (for caregivers). On the other hand, due to the particularity of cancer disease, patients are closely related to caregivers and interact with each other, so this study was conducted among patients but also caregivers. The aim of this study was to measure the comprehensive needs of cancer patients, and explore the possible factors associated with their needs.

## Methods

### Subjects and procedure

From April to October 2016, the cross-sectional study recruited participants involving cancer patients and caregivers from four tertiary hospitals in China by convenient sampling method: First Affiliated Hospital of Henan University of Science and Technology, Second Affiliated Hospital of Henan University of Science and Technology, Renmin Hospital of Henan Province, and Zhongxin Hospital of Luoyang. This study had been approved by the Human Research Ethics Committee of Henan Medical Association (2015–081025). Inclusion criteria for the cancer patients were: (1) over 18 years old; (2) being diagnosed with cancer; (3) currently receiving treatment or follow-up; (4) having the ability to read and write Chinese, and (5) willing to give their informed consents to participate in the study. The sample size was calculated by the following formula, $N={\left(\frac{U_{\alpha}\sigma }{\delta}\right)}^2$. In the formula, U_α_ is the U value corresponding to the testing level α, and σ is the total standard deviation, and δ is the admissible error. The total of 216 questionnaires were actually distributed and the response rate was 92.6% (200 of 216 were returned). The final number of completed cases was 200 cancer patients and 200 caregivers (See Table 1 for the basic characteristics of these participants).

**Table 1:** Demographic characteristics of 200 dyads of cancer patients and caregivers

	N (%)/Mean ± SD
Patient(N _1_ = 200)	
Age	54.87± ± 12.45
≥ 60	129 (64.5)
<60	71 (35.5)
Gender	
Male	96 (48.0)
Female	104 (52.0)
Ethnic	
Han	194 (97.0)
Minorities	6 (3.0)
Occupation	
No fixed occupation	35 (17.5)
worker	17 (8.5)
farmer	85 (42.5)
Cadre	22 (11.0)
retire	35 (17.5)
Others	6 (3.0)
Marital status	
married	188 (94.0)
unmarried	7 (3.5)
widowed	5 (2.5)
divorced	0 (0)
Education	
Primary school	53 (26.5)
Junior high school	71 (35.5)
Senior high school	53 (26.5)
College	23 (11.5)
Family monthly income per capita (yuan)	
<500	50 (25.0)
500~1000	63 (31.5)
1000~2000	48 (24.0)
>2000	39 (19.5)
Medical expense	
Public expense	11 (5.5)
Own expense	20 (10.0)
Medical insurance	169 (84.5)
Metastasis	
No	95 (47.5)
Yes	105 (52.5)
Type of disease	
Digestive system	90 (45.0)
Respiratory system	34 (17.0)
Blood system	4 (2.0)
Reproductive system	8 (4.0)
Osteosarcoma	14 (7.0)
Others	50 (25.0)
Time since diagnosis (year)	
<1	143 (71.5)
1~3	36 (18.0)
>3	21 (10.5)
Treatment measures	
operation	30 (15.0)
radiotherapy	10 (5.0)
chemotherapy	41 (20.5)
comprehensive therapy	119 (59.5)
Caregiver(N_2_ = 200)	
Age	44.26 ± 11.51
≥ 60	57 (28.5)
<60	143 (71.5)
Gender	
Male	98 (49.0)
Female	102 (51.0)
Ethnic	
Han	198 (99.0)
Minorities	2 (1.0)
Occupation	
No fixed	45 (22.5)
occupation	31 (15.5)
worker	63 (31.5)
farmer	29 (14.5)
Cadre	19 (9.5)
retire others	13 (6.5)
Marital status	
married	167 (83.5)
unmarried	30 (15.0)
widowed	2 (1.0)
divorced	1 (0.5)
Education	
Primary school	22 (11.0)
Junior high school	65 (32.5)
Senior high school	54 (27.0)
College	59 (29.5)
Family monthly income per capita (yuan)	
<500	32 (16.0)
500~1000	60 (30.0)
1000~2000	53 (26.5)
>2000	55 (27.5)
Number of caregivers	
1	119 (59.5)
2	63 (31.5)
3 or more	18 (9.0)
Together with patient	
Yes	181 (90.5)
No	19 (9.5)
Relationship with patient	
Spouse	84 (42.0)
Brother /sister	3 (1.5)
Child	88 (44.0)
Parent	18 (9.0)
others	7 (3.5)

**In the procedure,** written consent forms were signed by all participants after they fully understood the study. Anonymity and confidentiality were assured and participants were told that they could withdraw at any point without adverse consequences. Data was collected by the researchers with the unified guide language and data collecting procedure. The research team member checked the questionnaire for completion, and asked the participant to respond to each unanswered item. Patients’ comprehensive needs were assessed with Comprehensive Needs Assessment Tool in cancer for Patients (CNAT), along with the socio-demographic questionnaire.

### Measures

#### CNAT

The CNAT was initially developed and validated by Shim E.J. in a large scale involving 2661 cancer patients throughout Korea [23]. The Cronbach’s α for the scale was 0.97, and for subscales, it varied from 0.80 to 0.97. Principal component analysis resulted in an 7-factor structure explaining 64.2% of the total variance. For the first time, CNAT was translated into Chinese to assess the comprehensive needs of cancer patients in China. The Chinese version of CNAT has 59 items, a total of seven domains(Information, Psychological Problems, Health Care Staffs, Physical Symptoms, Hospital Facilities and Services, Social /Religious /Spiritual Support and Practical Support), assessing the comprehensive needs of cancer patients. Each item is scored from 0 to 3. “0” indicates “no need”, “1” indicates “low need”, “2” indicates “moderate need”, while “3” indicates “high need”. Standardization scoring method: Each dimension score = the practical score*100/items*3 [24]. The Cronbach’s α coefficient for the total CNAT score was 0.952, and 0.824–0.948 for the eight domains. Principal component analysis resulted in an 8-factor structure explaining 70.325%of the total variance [25].

#### Cancer patients general information questionnaire

This questionnaire contains 12 items, regarding socio-demographic and medical variables, such as age, gender, nationality, occupation, marital status, educational level, financial situation, with or without medical insurance, metastasis, disease type, time since diagnosis, and type of treatment.

#### Caregiver general information questionnaire

This questionnaire contains 15 items, regarding socio-demographic and medical variables, such as age, gender, nationality, occupation, marital status, educational level, financial situation, number of caregivers, whether to live with patients, relationship with patients. Diagnosis and disease stage were both retrieved from hospital information systems at the participating centers. Diagnosis of the cancer was divided into digestive system cancer/breast cancer/ respiratory system cancer/osteosarcoma/reproductive system or other types. Disease stage of the patient was divided into two types “cancer metastasis or no cancer metastasis”.

### Statistical analyses

All statistical analyses were performed with SPSS for Windows statistical software, version 20.0 s (IBM Corp., Armonk, NY, USA). All tests were bilateral, and *p* < 0.05 was considered as statistically significant.

The statistical description of the socio-demographic variables was carried out by frequency tables, means, and standard deviations. Domain scores of the comprehensive needs were calculated by averaging the score for each domain with subsequent linear transformation to a scale of 0–100 based on the EORTC scoring guideline [24]. For patients group, the mean differences in domain scores for different characteristics groups were compared by either one-way ANOVA or non-parametric analyses to see how these scores related to their socio-demographic and patients’ clinical characteristics (such as, the degree of disease, the treatment type and duration of cancer), depending on whether the data were normally or not normally distributed [26]. In this preliminary analysis, the total and each of the seven domains of the CNAT score were entered as dependent variables. The independent variables included age, gender, nationality, occupation, marital status, educational level, financial situation, with or without medical insurance, metastasis, disease type, time since diagnosis, and type of treatment**.** Second, multivariate regression analysis was performed to evaluate the related factors of the comprehensive needs and each domain need.

## Results

### Participant characteristics

The socio-demographic characteristics of cancer patients and caregivers were listed in Table 1. The mean age of cancer patients was 54.87 years old (SD = 12.45), and 52.0% were female. Most of the patients (84.5%) had the medical insurance. 52.5% had metastasis, and the treatment measures varied. Regarding the duration of cancer since diagnosis, 71.5% was less than 1 year. The main cancer types are digestive system (45.0%), breast cancer (25.0%), and respiratory system (17.0%).

### The comprehensive needs of cancer patients

Overall, the comprehensive needs of cancer patients was moderate, with the standardized total score 51.25 (SD = 9.69). The mean scores of each domain were listed in the Table 2. The highest score of need was for Health Care Staffs (78.35 ± 13.08), followed by Information (71.18 ± 17.39) and Hospital Facilities and Services (52.65 ± 13.35). Conversely, the lowest need was for Physical Symptoms (35.12 ± 16.68).

**Table 2:** The Comprehensive needs of cancer patients

		Information	Psychological Problems	Health Care Staffs	Physical Symptoms	Hospital Facilities and Services	Social /Religious /Spiritual Support	Practical Support	Standardized total score
N	Valid	200	200	200	200	200	200	200	200
	Missing	0	0	0	0	0	0	0	0
Mean value		71.1833	51.8333	78.3542	35.1250	52.6458	38.3000	39.0278	51.2514
SD		17.39153	11.86331	13.08062	16.68756	13.34700	12.46156	15.25653	9.69639
Minimum		0.00	0.00	4.17	0.00	0.00	0.00	0.00	1.69
Maximum		100.00	100.00	100.00	100.00	100.00	100.00	100.00	93.22

### Factors associated with the comprehensive needs of cancer patients

In the Table 3, significant differences were found in the comprehensive needs of cancer patients according to the various patients and caregivers characteristics. In this preliminary analysis, patients who were younger, female, with low family monthly income, at their own expense, more than 3 years after diagnosis, and with highly educated caregivers had higher score of CNAT. Then, in multivariate regression analysis, the nominal scale variable was converted to dummy variables. As a result, the factor with the greatest influence on the comprehensive needs(total score of CNAT) of cancer patients was “gender (female)”, followed by “medical insurance (at their own expense)” (Table 4). The overview of the risk factors of the comprehensive needs and each domain need of cancer patients was presented in Table 4. For the first domain(Information), the gender, medical insurance, education and the number of caregivers were significantly associated with this need. In the domain of Psychological Problems, the gender, medical insurance, income, and with or no metastases were related to this need. For Health-care staff, the gender, medical insurance and education showed a higher need. The age, gender and treatment measures were related to the need for Physical Symptoms. For the domain of Hospital Facilities and Services, income, treatment measures, the age of caregivers, showed a higher need. The gender, education, and with or no metastasis showed a higher need for Social and Religious / Spiritual Support. The gender, medical insurance, income, duration of disease, the age of caregivers, showed a higher need for Practical Support.

**Table 3:** Differences in patient’s needs by patient and caregiver characteristics

		Information	Psychological Problems	Health-care staff	Physical Symptoms	Hospital facilities and services	Social and religious/spiritual support	Practical support	Standardized total score
Patient									
Age	≥60	***65.23 ± 14.75***	***44.24 ± 13.15***	78.44 ± 13.56	***30.58 ± 7.57***	52.15 ± 13.47	38.18 ± 14.22	38.47 ± 12.52	***45.08 ± 9.23***
	<60	74.16 ± 15.72*	58.48 ± 12.11*	80.14 ± 12.48	38.26 ± 11.72*	53.28 ± 14.56	37.09 ± 15.56	39.57 ± 13.55	52.85 ± 10.52*
Gender	Male	69.13 ± 16.05	***45.35 ± 12.40***	***75.06 ± 13.68***	***30.40 ± 13.80***	52.71 ± 17.45	***35.69 ± 17.17***	***36.39 ± 12.41***	***42.63 ± 9.80***
	Female	72.07 ± 18.57	56.89 ± 13.10*	81.51 ± 11.95*	39.49 ± 18.52*	54.03 ± 13.13	42.05 ± 13.77*	41.46 ± 13.50*	49.13 ± 10.13*
Medical	Public	***65.83 ± 12.84***	***41.91 ± 15.83***	***72.77 ± 14.85***	***25.27 ± 16.33***	60.12 ± 10.57*	52.50 ± 27.88*	39.55 ***±*** 14.48	***45.02 ± 12.46***
	Own	75.00 ± 15.40*	53.33 ± 11.38*	80.95 ± 16.12*	46.94 ± 17.33*	***51.87 ± 11.64***	***40.66 ± 10.56***	44.71 ± 14.04*	58.48 ± 11.92*
	Insurance	***70.23 ± 17.25***	***42.49 ± 11.27***	***78.87 ± 11.95***	***33.90 ± 16.50***	***50.01 ± 12.83***	***36.11 ± 12.72***	***36.74 ± 14.86***	***46.73 ± 11.34***
Education	Primary	78.06 ± 16.63*	53.05 ± 12.27	79.96 ± 10.08	36.83 ± 19.47	51.51 ± 9.71	44.53 ± 10.49*	40.35 ± 13.55	52.32 ± 11.06*
	Junior	***70.45 ± 16.72***	48.65 ± 19.68	75.40 ± 13.63	33.55 ± 16.17	52.66 ± 14.02	***40.33 ± 13.61***	41.76 ± 12.93	***51.48 ± 11.13***
	Senior	69.45 ***±*** 18.71	52.01 ± 14.82	80.91 ± 13.77	33.95 ± 15.59	51.67 ± 14.61	***38.18 ± 12.25***	36.59 ± 13.00	***47.73 ± 9.54***
	College	***70.25 ± 17.60***	48.20 ± 19.81	77.93 ± 15.73	36.44 ± 13.70	52.18 ± 17.66	***35.49 ± 13.79***	40.40 ± 19.52	***49.62 ± 8.32***
Income	500	70.06 ± 17.64	42.53 ± 12.65*	82.75 ± 11.03	35.61 ± 19.29	52.32 ± 12.65	40.13 ± 10.17	42.22 ± 15.35*	56.11 ± 11.07*
	500~1000	68.46 ± 15.18	***36.45 ± 16.43***	81.34 ± 12.14	33.28 ± 14.63	***44.62 ± 10.44***	40.41 ± 13.77	***36.04 ± 11.61***	***46.47 ± 10.41***
	1000~2000	71.91 ± 17.41	38.44 ± 12.04	80.13 ± 13.75	36.10 ± 10.41	55.03 ± 10.66	40.49 ± 12.41	41.56 ± 13.82	***48.89 ± 11.25***
	2000	75.26 ± 20.44	37.01 ± 16.42	82.50 ± 15.49	33.46 ± 14.85	56.82 ± 17.06*	48.07 ± 16.27	37.92 ± 19.94	***50.63 ± 10.22***
Metastasis	No	71.09 ± 16.09	***47.02 ± 17.83***	79.32 ± 13.16	34.08 ± 15.65	54.23 ± 12.59	***38.53 ± 8.30***	38.89 ± 13.64	50.24 ± 11.57
	Yes	71.37 ± 18.46	52.41 ± 14.42*	77.54 ± 12.89	35.58 ± 17.39	52.46 ± 17.14	42.05 ± 10.51*	39.89 ± 26.03	52.06 ± 9.15
Time since diagnosis (year)	<1	***71.36 ± 16.38***	***51.71 ± 11.06***	***78.57 ± 12.93***	35.13 ± 17.24	53.04 ± 16.02	40.14 ± 16.06	40.16 ± 13.97*	***52.31 ± 9.59***
	1~3	***65.55 ± 19.62***	***45.64 ± 11.12***	***74.12 ± 15.55***	33.30 ± 14.90	52.33 ± 11.01	33.77 ± 17.20	***35.61 ± 22.15***	***46.18 ± 9.26***
	>3	80.66 ± 18.06*	59.83 ± 16.26*	84.79 ± 17.07*	35.83 ± 15.30	52.95 ± 10.40	41.66 ± 7.42	***33.88 ± 3.50***	56.38 ± 10.27*
Treatment	operation	71.83 ± 19.92	54.13 ± 10.44	79.54 ± 15.51	***36.24 ± 16.13***	***53.53 ± 15.43***	37.81 ± 10.24	39.69 ± 17.94	49.85 ± 13.26
	radiotherapy	73.77 ± 10.24	43.33 ± 7.63	75.88 ± 4.81	45.44 ± 10.03*	62.77 ± 14.69*	40.33 ± 14.03	38.88 ± 18.86	52.02 ± 9.06
	chemotherapy	72.43 ± 16.79	49.51 ± 13.47	76.56 ± 12.35	***36.62 ± 23.91***	***51.26 ± 15.78***	36.17 ± 13.20	37.23 ± 12.28	51.28 ± 9.45
	comprehensive	69.56 ± 17.07	51.38 ± 11.85	78.83 ± 12.98	***32.51 ± 13.94***	***43.70 ± 14.75***	40.02 ± 17.59	38.71 ± 15.08	50.05 ± 9.56
Caregiver									
Age	≥60	72.14 ± 14.32	50.85 ± 14.58	79.13 ± 10.28	34.48 ± 11.17	53.22 ± 10.65*	38.26 ± 14.44	***42.34 ± 12.67***	51.21 ± 10.04
	<60	69.42 ± 13.09	51.62 ± 11.13	77.07 ± 15.11	35.23 ± 10.54	***50.75 ± 17.49***	39.53 ± 10.74	35.36 ± 18.26*	50.03 ± 11.72
Education	Primary	70.54 ± 14.06	50.16 ± 17.51	***65.21 ± 12.38***	34.52 ± 16.36	***46.36 ± 10.68***	***25.75 ± 10.46***	39.82 ± 13.05	***47.27 ± 10.58***
	Junior	69.62 ± 12.05	50.25 ± 16.43	66.37 ± 14.55	35.37 ± 15.82	***45.15 ± 16.72***	***32.45 ± 14.26***	39.18 ± 14.78	***50.16 ± 10.43***
	Senior	72.83 ± 14.51	51.26 ± 10.44	72.51 ± 13.82	34.16 ± 14.52	***50.26 ± 14.72***	***35.11 ± 12.37***	38.56 ± 12.53	***50.25 ± 9.57***
	College	70.05 ± 16.42	52.82 ± 10.26	81.16 ± 12.52*	37.34 ± 14.46	58.57 ± 18.16*	48.45 ± 16.46*	40.06 ± 12.16	55.17 ± 10.25*
Number of caregivers	1	***64.04 ± 15.43***	49.27 ± 13.51	77.45 ± 12.23	***31.57 ± 13.25***	51.33 ± 14.73	39.46 ± 10.47	39.04 ± 17.25	50.64 ± 11.47
	2	74.24 ± 14.25*	52.58 ± 11.03	80.68 ± 12.23	***36.26 ± 17.94***	52.84 ± 13.42	38.71 ± 13.79	38.43 ± 14.37	50.21 ± 10.26
	3 or more	***81.82 ± 11.76***	51.36 ± 11.21	80.13 ± 11.28	40.53 ± 12.42*	52.92 ± 12.87	40.23 ± 11.98	40.21 ± 12.36	51.27 ± 9.29
Together with patient	Yes	70.11 ± 13.46	50.77 ± 12.29	78.84 ± 12.31	35.50 ± 17.13	52.87 ± 13.03	***33.77 ± 8.02***	39.24 ± 14.26	50.19 ± 10.03
	No	72.59 ± 11.23	52.96 ± 10.51	77.88 ± 19.18	36.51 ± 8.88	53.01 ± 12.68	42.74 ± 12.01*	40.33 ± 10.82	51.71 ± 9.36

*Note*: The bold and italics with * indicated the *p* <0.05

**Table 4:** Regression analysis for the comprehensive needs and each domain need and influencing factors

Dependent variable	Independent variable^a^	β	SE	p
Standardized total score of CNAT	Gender (Female)	0.423	4.308	< 0.001
	Medical insurance (at their own expense)	0.327	3.257	< 0.001
	Income (500)	0.236	4.336	0.015
	Caregiver Education (college)	0.303	2.205	< 0.001
Information	Age (Younger: <60)	0.201	3.535	0.003
	Medical insurance (at their own expense)	0.423	4.367	< 0.001
	Education (Primary)	0.218	3.264	0.021
	Number of caregivers(3 or more)	0.406	1.039	< 0.001
Psychological Problems	Gender (Female)	0.228	2.541	< 0.001
	Medical insurance (at their own expense)	0.103	1.346	0.006
	Income (500)	0.207	4.208	< 0.001
	Metastasis (YES)	0.114	2.335	0.034
Health-care staff	Gender (Female)	0.218	4.039	0.007
	Medical insurance (at their own expense)	0.135	2.357	0.018
	Caregiver Education (college)	0.323	1.268	< 0.001
Physical Symptoms	Age (Younger: <60)	0.236	2.553	< 0.001
	Gender (Female)	0.307	3.542	< 0.001
	Treatment (radiotherapy)	0.229	2.367	0.004
Hospital Facilities and Services	Income (2000)	0.359	2.158	< 0.001
	Treatment (radiotherapy)	0.225	3.346	0.011
	Caregiver age (≥60)	0.310	2.107	< 0.001
Social and Religious / Spiritual Support	Gender (Female)	0.327	2.038	0.001
	Income (500)	0.129	2.104	0.023
	Metastasis (YES)	0.402	3.238	< 0.001
Practical Support	Gender (Female)	0.405	3.208	< 0.001
	Medical insurance (at their own expense)	0.346	2.117	0.001
	Income (500)	0.228	1.259	< 0.001
	Time since diagnosis(<1 year)	0.309	2.037	< 0.001
	Caregiver age (≥60)	0.254	2.368	0.037

^a^Dummy variables: Gender (male) = 0, Age (≥60) = 0, Medical insurance (public) = 0, Income (500~1000) = 0, Education (college) = 0, Metastasis (NO) = 0, Time since diagnosis (1~3 year) = 0, Treatment (comprehensive) = 0, Caregiver age (<60) = 0, Caregiver Education (Primary) = 0

## Discussion

The present study is a questionnaire study including a relatively large sample of cancer patients with a range of cancer diagnoses at different times using the newly developed and validated questionnaire CNAT by specifically investigating the prevalence and risk factors of the comprehensive needs of cancer patients. Most previous studies addressing cancer patients have focused on the unmet needs [7, 11, 12, 16, 27–29] or a single kind of demand (such as supportive care demand [7, 8, 14, 15] and information need [17, 30, 31] or solely one kind of cancer(such as breast cancer, haematological cancer, lung cancer, Head and Neck Cancer, or cervical cancer). In addition, no previous studies have investigated the comprehensive needs of cancer patients (covering almost all aspects of demand) and their influencing factors. Therefore this study is a valuable supplement to the existing studies of cancer care. Moreover examining the comprehensive needs of cancer patients can inform the tailoring of interventions or supports to the specific needs, in order to enhance their quality of life [32].

Regarding the cancer patients’ status of need, one finding in this study was that the overall need of cancer patients was at medium upper level in agreement with previous findings [8, 33]. Among the seven domains of needs, the highest score was the need for Health Care Staffs, followed by the need for Information and the need for Hospital Facilities and Services, which was to some extent in accordance with the preview studies including the unmet needs for health system, information, and patient support [34, 35]. Most patients have always wanted a hospital staff who can talk about all aspects of their condition, treatment, and follow-up, with remaining unmet needs addressing mostly desire for information [36]. Our finding that the lowest score was the need for Physical Symptoms domain (35.12 ± 16.68), was inconsistent with a systematic review [37], which found the most frequently reported unmet needs were those in the activities of daily living domain, followed by psychological, information, psychosocial and physical domain, suggesting that the Chinese cancer patients were more inclined to focus on the needs of other aspects than the physical symptoms.

Our study also indicates that subgroups of cancer patients experience different types of needs, with the predictors of reporting some unmet need for help varying according to the domain examined. Sociodemographic characteristics were associated with comprehensive needs of cancer patients. In general, comprehensive care should be given to these patients who were female, with low family monthly income, at their own expense and with highly educated caregivers.

The present study indicated that gender is a relevant factor for being at risk of having more comprehensive needs and that female cancer patients are more likely to have unmet needs than male patients, especially need for Psychological Problems [38]. Similar findings have been confirmed to explain this difference, such as the female gender being associated with increased anxiety and/or depressive disorders [39]. Due to gender factor, female cancer patients are more psychologically affected than male patients and think more about many aspects of the disease [40], which in turn leads to higher demand, especially for Psychological Problems [34], Health-care staff, Physical Symptoms, Social and religious / spiritual support and Practical Support. Therefore, female cancer patients should be monitored more carefully and may be a target population for providing more intensive care.

Owing to the high cost of treatment, low-income patients with cancer experienced high levels of unmet needs across a wide range of psychosocial needs, such as the practical, health professional and cancer-related information, which had been confirmed by previous studies [41]. Likewise, many patients at their own expense also face economic pressure because of no insurance, which may lead to high need for psychosocial support, especially financial support. Financial stress and strain due to cancer, have been shown to be associated with adverse psychological outcomes in breast and prostate cancer patients [42, 43] and thus the intersection with psychological unmet needs is not unexpected. Accordingly, to bridge the gap between increasing cancer patients needs and limited resources, the development of interventions designed to aid in cancer patients screening and resource identification should be suggested.

Our data may suggest that patients with lower educational levels or with highly educated caregivers reported higher comprehensive needs. These findings correspond with the results of the previous studies [44, 45]. It may be that cancer patients with lower educational levels reported a greater need for transportation services, treatment near their house and help with economic burden, as well as help with worries that they would become a burden to others. On the other hand, the educated caregivers have better access to health facilities and information about cancer care, whereas they need more information about how to provide better care of the patients.

Analysis of influencing factors of needs in different domains indicated special consideration may need to be given when planning the care of cancer patients of different characteristics; such as focusing on providing information for younger, at their own expense, less educated patients and with three or more caregivers. For patients who were female, at their own expense, low income and with metastasis, necessary psychological care may need to be provided. And for female patients who were at their own expense and with highly educated caregivers, health-care staffs should communicate more with them to increase their sense of trust and security. Younger, female patients and perceived with radiotherapy showed a greater need for physical symptoms, so more interpretation and guidance should be provided to them. For patients with high income, perceived with radiotherapy, with older caregivers, the provision hospital facilities and services should be paid more attention to. Social and religious / spiritual support should be provided for female patients, low educated and with metastasis. Last, practical support should be provided for female patients, with medical care at their own expense, low income, diagnosed less than a year and with older caregivers, such as transportation services or financial support. These findings suggest that interventions that mobilize social and health care support may, therefore, provide multilevel benefits across the cancer trajectory according to the different characteristics of patients [38].

### Limitation

Even though the present study provides important information of comprehensive needs of cancer patients, it has some limitations including the cross-sectional design, sample bias, and the small sample size. First, participants in the study were drawn mainly from hospital patients who were receiving or had received treatment for their disease. This selection bias may have implications for the findings of the study to some extent. The findings therefore are likely to relate to the experiences of a sub-group of individuals who may be fitter than those who receive hospital treatment alone. Second, there is a risk of bias across the study to detect potentially vulnerable subgroups as being at risk because these subgroups are too small in numbers to be quantitatively analyzed as potential predictors of need. While the present study is able to elicit specific needs, it has small biased sample and we cannot generalize these findings to all cancer survivors. Further studies should be undertaken to confirm the present findings.

## Conclusions

In view of our findings, we conclude that cancer patients experience high levels of needs for health-care staff and information, and the different needs are closely related to their sociological characteristics. Understanding the comprehensive needs of patients with cancer is essential to improving the care and outcomes. These findings highlight the importance of providing adequate support to address the diversity of patients needs, thereby ensuring sustainable provision of care and support to the patients. Future studies should incorporate patients’ care interventions to better understand those individual situations and how they may influence the outcomes of cancer patients.

## Data Availability

The data used for this study is confidential and cannot be made public. Individuals interested in obtaining specific data may contact Dr. Hongyun WANG (zxs1214@163.com).

## Abbreviation

CNAT: Comprehensive Needs Assessment Tool in cancer for Patients
